# Surgical Management of Brain Metastases in the Perirolandic Region

**DOI:** 10.3389/fonc.2020.572644

**Published:** 2020-10-26

**Authors:** Fuxing Zuo, Ke Hu, Jianxin Kong, Ye Zhang, Jinghai Wan

**Affiliations:** ^1^Department of Neurosurgery, National Cancer Center/National Clinical Research Center for Cancer/Cancer Hospital, Chinese Academy of Medical Sciences and Peking Union Medical College, Beijing, China; ^2^Department of Radiation Oncology, National Cancer Center/National Clinical Research Center for Cancer/Cancer Hospital, Chinese Academy of Medical Sciences and Peking Union Medical College, Beijing, China

**Keywords:** brain metastases, eloquent areas tumors, surgical treatment, individualized approach, perirolandic mass lesions

## Abstract

Brain metastases (BM) are the most frequent intracranial tumors, which may result in significant morbidity and mortality when the lesions involve the perirolandic region. Surgical intervention for BM in the perirolandic region is still under discussion even though prompt relief of mass effect and avoidance of necrosis together with brain edema may not be achieved by radiotherapy. More recently, several researchers attempt to evaluate the benefit of surgery for BM within this pivotal sensorimotor area. Nevertheless, data are sparse and optimal treatment paradigm is not yet widely described. Since the advance in intraoperative neuroimaging and neurophysiology, resection of BM in the perirolandic region has been proven to be safe and efficacious, sparing this eloquent area while retaining reasonably low morbidity rates. Although management of BM becomes much more tailored and multimodal, surgery remains the cornerstone and principles of resection as well as indications for surgery should be well defined. This is the first review concerning the characteristics of BM involving the perirolandic region and the current impact of surgical therapy for the lesions. Future perspectives of advanced neurosurgical techniques are also presented.

## Introduction

The perirolandic region is essential for neurological functions, supporting motricity, and sensitivity of trunk and extremities (1, 2). Notably, brain metastases (BM), the most common intracranial tumors (3), tend to be located in the eloquent areas such as the perirolandic region where sensorimotor function is often disrupted (1, 4–7). With advances in neuroimaging, neurophysiology, and neurosurgical techniques, patient-tailored surgery has become the pivotal strategy in multimodal treatment paradigms of BM. However, approaching BM in the perirolandic region remains a challenge because there may be a risk of new permanent neurological deficits resulting from impairment of cortical or subcortical structure after resection of the tumors which infiltrate into the surrounding sensorimotor areas (4, 7–11).

Controversy exists regarding optimal treatment for patients with BM within the perirolandic region (7, 11–22). Less-invasive therapies including whole-brain radiation therapy (WBRT) or stereotactic radiotherapy (SRT) has still been preferred although relief of the mass effect was always delayed and patients often suffered from adverse events produced by radiation (5, 12, 13, 17, 20, 22, 23). Alternatively, several studies have suggested that optimal resection could promptly reduce mass effect, relieve neurological symptoms, provide pathological diagnosis, and improve local tumor control (4, 6, 10, 16, 18, 24–26). To the best of our knowledge, there are only a few reports with regard to surgical treatment of BM involving the perirolandic region (4, 6, 16, 18, 24, 26, 27), while no literature reviews have been performed, leaving the optimal treatment paradigm unresolved.

This review summarizes the impact of surgery on the multimodal management of BM in the perirolandic region. Close attention has been paid to the indications for surgery, the principles of resection, and the individualized surgical approaches. Future minimally invasive and multimodal therapies such as laser interstitial thermal therapy (LITT) are discussed, which may lead to new paradigm for management.

## Anatomy

**Figure 1:**
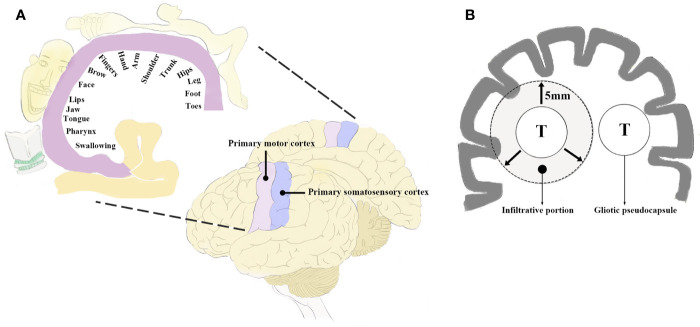
**(A)** Diagram of the perirolandic region illustrating the primary motor cortex, primary somatosensory cortex, and the classic homunculus. The areas of sensorimotor cortex supporting motricity and sensitivity are inverted and disproportionate based on the sensorimotor patterns (fine or gross) as depicted in the coronal planes. **(B)** Schematic illustration exhibiting the infiltrative growth pattern of BM which may extend to 5 mm beyond the gliotic pseudocapsule. T, tumor; BM, brain metastases.

The perirolandic region, also known as central lobe (1) or paracentral area (6), is one of the most eloquent areas of the brain, which consists of pre- and postcentral gyrus, central sulcus, and the paracentral lobule (1, 2). The lateral surface of the perirolandic region includes the precentral and postcentral gyri divided by the central sulcus and limited anteriorly by the precentral and posteriorly by the postcentral sulcus. The medial surface extends into the interhemispheric fissure to form the paracentral lobule which is limited by the paracentral sulcus anteriorly, the ascending ramus of the cingulate sulcus posteriorly, and the cingulate sulcus below. When operating in the perirolandic region, it is important to remain constantly aware of the arterial supply as well as venous drainage because neurological deficits following tumor resection is more frequently due to arterial or venous infarction than to cortical or subcortical structural impairment (28). The central artery arising from middle cerebral artery (MCA) supplies a larger part of the central lobe than any other arteries. When performing transsulcal approach, care must be taken to avoid coagulation of any branches arising from the central artery within the sulcus, which may lead to motor weakness. The central vein usually drains the largest portion of the central lobe. Although there are anastomoses located at the terminal ends of veins just proximal to the superior sagittal sinus (SSS), sacrifice of major bridging veins such as central vein or precentral vein should be avoided. Obliteration of the cortical veins from the perirolandic region may cause severe contralateral hemiparesis prominent in the lower limbs (1, 29). Ribas et al. (2) considered the perirolandic region as a single lobe because the gyri, sulcus and subcortical white matter such as corticospinal tract (CST) shared the common patterns in configuration and sensorimotor function. The fibre tracking technique could reconstruct the white matter trajectories, particularly the CST and thalamocortical radiations which connected the sensorimotor cortex to the peripheral nervous system. The perirolandic region identification based on functional magnetic resonance imaging (fMRI) and direct electrical brain stimulation demonstrated its role as sensorimotor center and disproportionate arrangement of different body parts in an inverted fashion in this area called homunculus (1, 30) (**Figure 1A**). The different parts of the body were represented in approximately bottom-to-top order, while the cortical areas supporting fine movements were disproportionally larger than areas supporting more gross movements (1). An understanding of anatomy is important to create a road for approaching lesions in the perirolandic region.

## Epidemiology

BM are the most common neoplasms in the central nervous system (3), with an approximately 10–40% of all cancer patients ultimately developing the condition (3, 4, 31–33). These tumors mostly originate from melanoma, lung and breast cancer (3, 9, 31). Currently, the rising incidence of BM is due to an aging population, effective targeted therapies for systemic disease, and advances in sensitive diagnostic tools (4, 31, 34). It is well known that diagnosis of BM portends a poor prognosis, with median survival time varying from 1 to 6 months in untreated patients (3, 9, 21, 34–38).

BM have been estimated to occur as many as 10 times more frequently than primary brain tumors (3, 33, 34). Although the distribution of single BM predominates in parietal lobe (50.98%) followed by frontal lobe (25.49%) (33), the prevalence of BM involving the perirolandic region has not yet been clarified. Yoo et al. (32) analyzed 51 patients (54.26%, 94 metastases in total) with lesions in eloquent locations. In a cohort of 1,033 BM, there were 288 tumors (27.88%) in eloquent areas (31). However, these retrospective analyses were all single institutional studies which reviewed BM in or directly adjacent to eloquent areas including language, vision, or sensorimotor regions. Krieg et al. (18) reported a series of 206 metastases including 56 (27.18%) in the eloquent motor area, which probably exhibited reliable data with regard to the incidence of the tumors.

## Invasion Patterns

BM were usually regarded as sharply delimitated within brain parenchyma (8, 32). Neuro-radiological findings presented well-demarcated contrast-enhanced areas comparing with that of primary malignant brain tumors. In fact, the infiltrative growth pattern has been identified, exhibiting a tongue-like expansion into the surrounding tissue (8, 35, 39, 40). Recently, 5-aminolevulinic acid (5-ALA) was administrated in detecting tumor border to adjacent brain tissue during BM resection and found almost 40% metastases as well as tumor beds depicted fluorescence (39, 41). There were three different invasion patterns of BM (8, 35): **i)** well-demarcated border to surrounding white matter; **ii)** perivascular protrusion from main tumor mass; **iii)** diffuse infiltration of single cells into surrounding brain parenchyma (**Figure 1B**). Baumert et al. (42) reported 63% BM displayed evidence of infiltration in an autopsy study. Siam et al. (43) performed clinical study and corroborated the above autopsy findings. Sundaresan et al. (8, 32, 35, 39, 40, 44) suggested the infiltrative tumor cells seldom extended to more than 5 mm beyond the gliotic pseudocapsule.

The invasion patterns might assume major prognostic significance (8). Pure circumferential stripping of BM was sometimes insufficient in achieving disease control, and unexpected residual tumors might result in local progression (9, 32, 43, 45). The incidence of local tumor progression after BM resection has been reported up to 40% (31, 32, 46). Moreover, 10–34% patients had recurrence in surgical cavity 1 year after treatment even though they underwent complete removal together with adjuvant radiotherapy (23, 32, 38, 45, 47). In a recently published prospective EORTC 22952-26001 study, 27% patients receiving postoperative WBRT and 58% patients without adjuvant therapy suffered from local tumor recurrence (22).

BM tend to grow in the cortical-subcortical junction and significantly displace the sensorimotor cortex or the subcortical white matter tracts if located in the perirolandic region (4). Resection could be conservative in this area because the tumors probably infiltrate and disrupt the surrounding critical structures in addition to displacement of them. Consequently, the infiltration into the adjacent sensorimotor areas may play a pivotal role in local recurrence after gross total resection and must challenge therapeutic indication because of concern for cortical or subcortical structure impairment.

## Current Strategies

The treatment of BM is more tailored and multimodal, including surgical, radiation, and systemic therapies (9, 35). Personalized therapeutic paradigms mainly depend on decisions provided by multidisciplinary oncological specialists. It is widely accepted that surgical resection is ideally followed with adjuvant radiotherapy (13, 21, 23, 35, 48, 49). SRT to postoperative surgical cavity has increasingly replaced WBRT as the standard of practice following the resection of tumors (12, 13, 21, 48). The rate of local control at 12 months was found to be 83.7% with low rate of radiation necrosis (6.9%) in a meta-analysis, and patients receiving fractionated SRT had better local control than those treated with single fraction SRT (87.3 vs 80.0%) (48). Although the adjuvant hypofractionated SRT resulted in high tumor local control rates with low toxicity, prompt relief of mass effect and symptoms could be delayed (9, 13, 21, 35).

Surgical intervention for BM in the perirolandic region has still been a matter of debate, since there may be a potential risk of new permanent neurological deficits during the postoperative period (4, 19, 26). Less-invasive therapies such as WBRT and SRT have been previously preferred in several institutions (5, 20, 23, 38). Luther et al. (5) reviewed patients underwent SRT for BM located in the motor cortex and indicated that worsening of motor function occurred in less than 20% of patients. They suggested radiation provided both tumor control and a low risk of producing neurological deficits (5). Williams et al. (23) found 11% of patients treated with SRT developed new motor deficits for lesions located in the perirolandic region. Another study focusing on treatment of BM in the precentral gyrus suggested 26% of patients suffered new neurological deficits after SRT, including new onset refractory epilepsy (27). More recently, Pintea et al. (19) pointed out the overall incidence of complication and improvement ratio of pre-treatment motor deficits were 35.7 and 17% in the SRT group, comparing with 25 and 54% in surgical group, respectively. It is clear that the adverse events produced by radiation cannot be negligible. Radiotherapy may have several disadvantages of its own in the BM therapy (5, 12, 13, 17, 19, 20, 23, 27, 46, 50, 51): **i)** cannot promptly relieve the symptoms or even develop new deficits caused by mass effect, brain edema, or hydrocephalus; **ii)** cannot provide definite histological diagnosis which may facilitate the chemotherapy or targeted therapies; **iii)** has been generally limited to lesions which are less than 3cm in maximal diameter; **iv)** may cause symptomatic radiation necrosis with an incidence of 5%.

## Advances in Technology

The introduction of 5-ALA fluorescence-guided resection has significantly improved local tumor control for gliomas (41, 52). Recently, 5-ALA was administered prior to surgery for BM to detect tumor which infiltrated adjacent brain tissue (9, 16, 39, 41). But some of these studies met with disappointing results, which showed that the tumor border could only be verified after circumferential resection in less than 40% patients (39, 41). Alternatively, fluorescein sodium (FS) has been used to distinguish intracranial tumors from brain parenchyma since 1948 (53). More recently, Schebesch et al. (54) reported a high rate of FS uptake (90%) in BM, resulting in significant increase in the number of patients receiving gross-total resection (GTR) (83.3%). Höhne et al. (55) performed fluorescence-guided resection of BM with FS. Fluorescein was considered helpful in distinguishing tumors from viable tissue in 95% of patients. Technical adjuncts including the intraoperative ultrasonography and neuronavigation were used only for tumor localization but not for resection control. Similar to what other authors have described (55–57), the visibility of intraoperative ultrasonography remained clear during the surgical procedure and was helpful in tumor resection in 86.67% (13/15) of cases (56). There were few reports referring to combination of ultrasound and fluorescence-guided BM resection. More recently, Barbagallo et al. (57) investigated the safety and extent of BM resection. The fluorescence-guided resection and neuromonitoring including intraoperative ultrasonography and computed tomography (CT) were used in cased of lesions allocated in eloquent areas. Fluorescence was negative in 2 cases (25%), while the presence of residual tumor confirmed by intraoperative CT scanning was often less than that anticipated by ultrasound, demonstrating that intraoperative ultrasonography might generate false positives (57). Histologically, clean surgical margins have been confirmed by intraoperative fresh-frozen sectioning in the Yoo study (32). Frozen biopsy samples were obtained from multiple sites, including anterior, posterior, medial, lateral, superior, and inferior walls of the surgical cavity (8). If reports were positive for tumor cells, more biopsy samples were collected at or near the suspicious areas (8, 32, 39). Although the clean tumor border could be verified, extra surgical time was needed to perform the pathological analysis. The benefit of fluorescence-guided resection and fresh-frozen sectioning still needs to be evaluated in future prospective trials.

**Figure 2:**
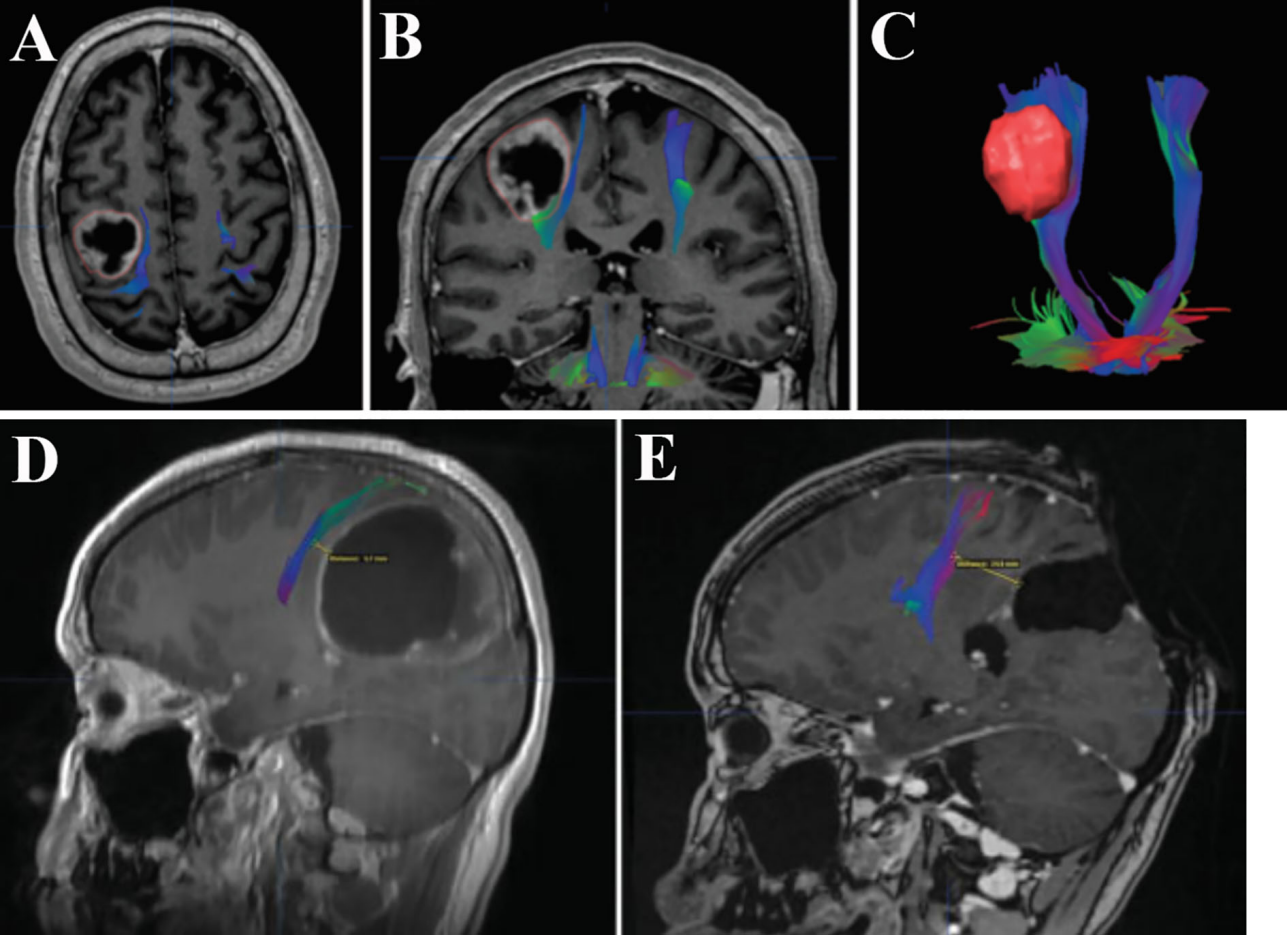
Merged DTI sequence and contrast T1-weighted MRI revealing the proximity between BM and the CST. A cystic lesion with peripheral contrast enhancement allocated in the perirolandic region was depicted in the axial **(A)** and coronal **(B)** planes. The proximity was noted between the deep surface of the tumor and the descending fibers of the CST **(B)**. The 3D reconstruction of the CST and volumetric reconstruction of the lesion showed the distortion of the motor pathways **(C)**. Total removal of another large cystic tumor which significantly displaced the CST **(D)** was confirmed by postoperative MRI **(E)**. Notably, the distance between surgical cavity and the CST increased from 5.7 to 24.9 mm, suggesting the effect of initial deformation accompanied by intraoperative brain shift. DTI, diffusion tensor imaging; MRI, magnetic resonance imaging; BM, bran metastases; CST, corticospinal tract. **(A–C)** Modified from Sanmillan et al. (4). **(D, E)** Modified from Krivosheya et al. (60).

Together with the advances in neuroimaging and neurophysiology, fMRI helps to examine and visualize sensorimotor cortex and white matter tracts, which could facilitate preoperative planning as well as navigated resection of BM affecting the perirolandic region (10, 18, 58, 59). The diffusion tensor imaging (DTI) sequence with 3D reconstruction might definitely delineate the relationship between tumor and the sensorimotor pathways (**Figure 2**), which is useful in exploring individualized surgical approaches (4, 10, 60, 61). Sanmillan et al. (4) performed resection of BM allocated immediately anterior to the CST (**Figures 2A–C**) *via* dissection of the precentral sulcus. Total removal of the tumor was achieved, while the patient showed improvement in the initial symptoms. The perirolandic lesion frequently causes critical displacement and distortion of the pyramidal tract or the thalamocortical radiations as being depicted by DTI (4, 6, 16, 18, 60). Bobek-Billewicz et al. divided the patterns of white matter tract alterations into 5 types based on DTI (62): i) untouched; ii) deviated; iii) oedematous; iv) infiltrated; v) destroyed. They found that most BM deviated the white matter tracts which could be oedematous. The fractional anisotropy (FA) values were significantly lower and the apparent diffusion coefficient (ADC) values were significantly higher within the precentral gyrus and CST in patients with neurological deficits than ones without them. Although preoperative DTI parameters might be important for fibre tracking, there were some reports referring to the limitations in localizing perirolandic region due to tumor metabolism and edema (15, 63). Furthermore, neuronavigation might be less accurate because images obtained prior to surgery could be subject to brain shift (64, 65). Following dural opening, cerebrospinal fluid (CSF) drainage, and tumor resection, brain shift might be as large as 2.4 cm (4). The safest non-eloquent approach to the lesion without disturbing the adjacent sensorimotor tracts was established by employing intraoperative MRI including DTI data (**Figures 2D, E**) (26, 60, 61, 66). D’Andrea et al. (61) performed intraoperative DTI for tractography after the dural opening to correct the potential brain shift. The white matter bundle containing the CST was visualized in all patients following correcting an average error of 0.79 ± 0.25 mm, and the overall data illustrated 75% of cases in contact and/or involving the motor tracts. Only 1 patient presented transient weakness of left extremities which dramatically improved 1 month later and then disappeared after 3 months.

**Figure 3:**
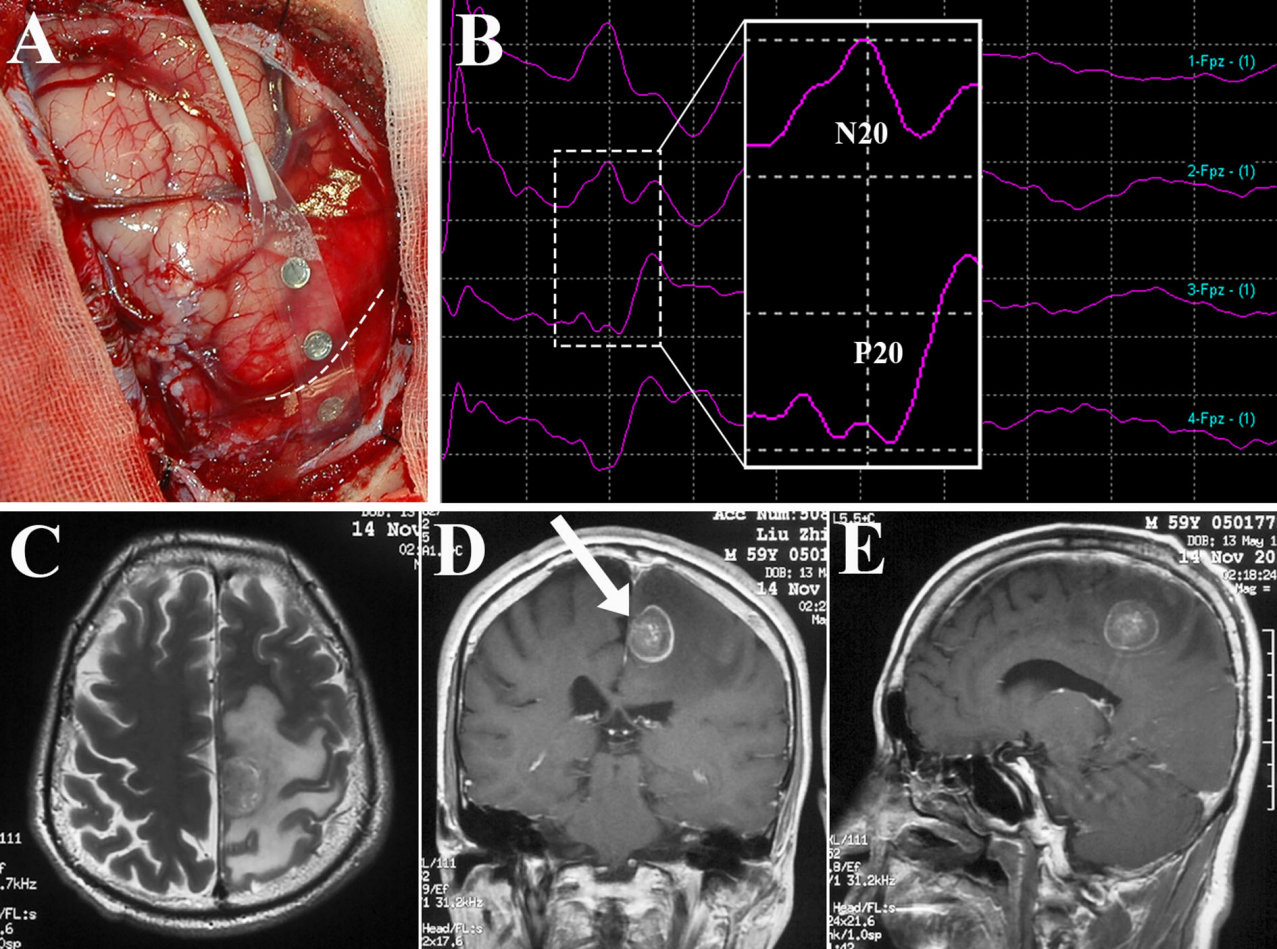
Identification of the central sulcus and sensorimotor cortex using brain mapping. Subdural electrode strip was placed over the perirolandic region **(A)** to record SEPs, and N20-P20 phase reversal **(B)** gave information about the site of the central sulcus (dotted line in A). After direct motor cortex mapping, BM allocated within the left paracentral lobule as depicted in axial T2-weighted MRI **(C)** and contrast sequence in the coronal **(D)** and sagittal planes **(E)** was removed *via* the contralateral transfalcine approach (arrow in **D**). SEPs, somatosensory evoked potentials; MRI, magnetic resonance imaging.

Since Penfeld and Boldrey first described brain stimulation techniques (67), intraoperative brain mapping combined with intraoperative neurophysiological monitoring (IONM) have permitted the delimitation of cortical sensorimotor areas and subcortical functional pathways, and evolved to become the gold standard for preserving neurological function during BM resection (4, 68). The electrode strip is placed over the sensorimotor cortex to identify the central sulcus, precentral gyrus, and postcentral gyrus by recording the N20-P20 phase reversal somatosensory evoked potentials (SEPs) and performing brain mapping (**Figure 3**). Thereafter, corticotomy can be performed within the nonfunctional area which is determined by cortical motor mapping (4, 25, 67–69). During resection of BM in the perirolandic region, cortical as well as subcortical electrical stimulation are continuously produced using electrode strip and monopolar electrode respectively to assess the motor response and demarcate the area where proximity to the CST is suspected (68, 69). Finally, when resection is finished, brain mapping could evaluate the functionality of sensorimotor pathways. Obermueller et al. (70) performed direct cortical motor evoked potentials (MEPs) aiming on preservation of motor tracts. There was no real false negative case, but 11 cases (19.64%) were categorized as false positive ones when MEPs decline >50% was considered, resulting in high rate of subtotal resection (STR). Thus, an amplitude decline >80% was recommended. Sanmillan et al. (4) combined the transcranial, cortical, and subcortical stimulation to double check the functional integrity of sensorimotor pathways. Intraoperative partial motor seizure occurred in 1 case and was terminated by cold Ringer’s lactate. Postoperatively, the patient received anticonvulsant medication and was asymptomatic without any further seizures, while other 4 patients (12.12%) developed a transitory worsening in their paresis, who recovered within 3 months. Recently, high-frequency (HF) stimulation was adopted during resection of tumors within perirolandic region for motor mapping with lower rates of stimulation-induced seizures compared to traditional low-frequency (LF) stimulation mapping (71, 72). Bander et al. (72) identified motor cortex using HF bipolar stimulator. Continuous cortical MEPs monitoring was elicited throughout the tumor resection without any decrement in all cases (100%), but LF bipolar stimulation was successful at eliciting MEPs only in 30.77% of the cases. Notably, there was no intraoperative seizures occurred in group of HF bipolar stimulation. Apart from primary motor cortex, the functional integrity of sensory tracts is hard to be evaluated, and further studies of mapping techniques which have improved sensitivity as well as specificity are still required.

## Neurosurgical Management

With advances in neuroscience and technologies, surgery remains the cornerstone in BM treatment because of prompt relief of the mass effect and resultant clinical symptoms (9, 21, 26, 31, 32, 35, 46, 73). There have been numerous publications concerning surgical treatment of BM located in the sites of high eloquence including premotor, motor, sensory, speech, vision centers, and so on (39, 40, 74, 75), whereas only a few, until now, have particularly focused on the tumors directly involving the perirolandic region. Neither prospective randomized controlled trials nor systemic reviews have been performed, leaving the optimal treatment algorithm largely unresolved. Weil et al. (27) first specifically analyzed a series of 17 patients who underwent resection of BM within the primary motor cortex, and demonstrated that excision of BM could preserve or improve neurological function with meaningful increases in quality of life and survival time. Rossetto et al. (16) performed the first study on resection of BM in the perirolandic area by means of IONM including cortical mapping, and showed that 90% of patients improved or remained stable after surgery. However, clinical follow-up of 1 month was definitely short not to define and assess the postoperative outcomes accurately. More recently, Sanmillan et al. (4) reported the largest series of BM patients in the perirolandic region receiving surgical treatment. The patient-tailored IONM was also performed to achieve gross total resection in 93.9% of patients, total recovery of neurological deficits 3 months after surgery, and the mean survival time of 24.4 months. According to the retrospective studies schematically illustrated in **Table 1**, removal of BM in the perirolandic region is safe and effective with good expectation for long-term local control (4, 6, 7, 16, 18, 19, 24, 26, 27, 39, 76). Therapeutic decision should rely on several factors including tumor characteristics, patient’s condition, and status of systemic disease (19, 21).

**Table 1:** Reports of patients who underwent resection of BM in the perirolandic region.

References	No. of Patients	GTR Rate	Transient Neurological Deficits	New/Worsened Permanent Neurological Deficits	Postoperative KPS≥70	Local Recurrence Rate	Mean OS (Months)
Weil and Lonsen (27)	17	100% (17)	3 (17.65%)	1 (5.88%)	94.12% (16)	0%	10.3
Shinoura et al. (24)	11	54.55% (6)	6 (54.55%)	1 (9.09%)	–	–	–
Walter et al. (6)	20	95.00% (19)	2 (10.00%)	1 (5.00%)	60% (12)	–	–
Kamp et al. (39)	12	100% (12)	2 (16.67%)	0	–	–	–
Kellogg and Munoz (26)	17	100% (17)	1 (5.88%)	1 (5.88%)	82.35% (14)	–	–
Krieg et al. (18)	37	89.19% (33)	10 (27.03%)	5 (13.51%)	–	–	–
Rossetto et al. (16)	47	93.62% (44)	17 (36.17%)	5 (10.64%)	89.36% (42)	–	–
Sanmillan et al. (4)	33	93.94% (31)	6 (18.18%)	0	75.76% (25)	–	24.4

BM, brain metastases; GTR, gross-total resection; KPS, Karnofsky Performance Scale; OS, overall survival.

### Surgical Indications

The recursive partitioning analysis (RPA) classification provided by the Radiation Therapy Oncology Group (RTOG) is graded based on age, functional status characterized by the Karnofsky Performance Score (KPS), and control of systemic disease, which facilitates to select appropriate patients for surgical treatment (14). Guideline papers suggest that class I or II patients who suffer larger BM (>3 cm in diameter) or those developing significant mass effect may have better outcomes with surgical resection followed by radiotherapy which can offer increased local control rates (9, 13, 17). Notably, even the small lesions (<1.5cm in diameter) within the perirolandic region may present acute neurological deterioration and represent a surgical indication (21). Nevertheless, the indications for surgery in the setting of BM involving the perirolandic region have been less well defined.

With the advances in therapeutic options for systemic cancer and improvement of neurosurgical techniques as well as instruments, more patients with BM in the perirolandic region may benefit from aggressive surgical excision which could be performed in the following situations as depicted in **Table 2** (9, 21, 27, 74). In general, all efforts must be directed toward the avoidance of permanent neurological deficits postoperatively (4, 7, 14, 27, 34), because deteriorated functional status could deprive the patients of the chance for adjuvant therapies, significantly affecting overall survival.

**Table 2:** Indications for surgery of BM in the perirolandic region.

Multiple factors	Surgical indications
Therapeutic	Symptomatic lesion with brain edema Cystic or necrotic lesion Tumor hemorrhage requiring immediate relief Lesion with mass effect or associated hydrocephalus
Diagnostic	No known primary cancer Potential differential diagnosis Suspected symptomatic brain radionecrosis
Strategic	Potentially eligible for identifying new molecular targets and associated therapies
Prognostic	Stability of systemic disease Estimated overall survival time >6 months

BM, brain metastases.

### Principles of Tumor Resection

The oncological idea that en bloc excision, defined as circumferential stripping of lesion along the brain-tumor interface without violation of its capsule may obtain local disease control and improve the survival time has been widely accepted (4, 9, 31, 32, 35, 40). Previous reports suggested that en bloc resection of BM was associated with lower rates of leptomeningeal dissemination (77, 78) and local recurrence (79) than piecemeal resection. But en bloc resection of BM was not always achievable particularly in eloquent areas (13, 47, 75). Recent results supported this technique as feasible when tumor involved the functional brain regions (4, 31, 35). Sanmillan et al. (4) performed en bloc microsurgical resection of BM located in the perirolandic region with the help of intraoperative mapping techniques. The authors indicated that the patient-tailored fMRI-guided approach combined with IONM could spare the sensorimotor areas, thereby avoiding new permanent neurological deficits.

As previously described, BM might display an infiltrative growth pattern, extending towards surrounding brain parenchyma (8). Therefore, some authors designed the technique of microscopic total resection (MTR) [also known as supramarginal resection (39)] which included removal of apparently normal-looking surrounding brain tissue to a depth of 5 mm (confirmed by neuronavigation) after GTR of the tumors (32, 39, 40). Kamp et al. (39) provided the first data on the outcomes of supramarginal resection of 12 BM located within the perirolandic region using awake mapping, which revealed that none of the patients suffered from new permanent neurological deficits whereas only 2 displayed temporal disturbances. In addition to MTR, clean surgical margins have been confirmed with cavity biopsies sent for intraoperative fresh-frozen sectioning (32). However, most investigators have preferred intraoperative brain mapping and subcortical stimulation rather than MTR because continuous feedback of neuronal fiber pathways might prevent permanent neurological deficits (4, 9, 63, 80, 81). They suggested that supramarginal resection could not be achieved because intraoperative sub-cortical stimulation revealed new neurological deficits when performing MTR in the eloquent areas. Although eloquently situated BM can be eligible for supramarginal resection, further studies are still necessary to evaluate complication rate, local tumor control, and overall survival.

### Individualized Surgical Approaches

Regarding BM at the sensorimotor strips, the central sulcus can be dissected to minimize brain retraction and contusion (11, 32, 76). Then the corticotomy was preferably established in the depth of the central sulcus overlying the tumor (32). Lee et al. (11) chose to dissect the central or precentral sulcus rather than directly incised in the gyrus based on the shortest distance to the tumor border and showed favorable outcomes. But as previously described, the necessity of DTI fiber tracking needs to be emphasized since it is able to exhibit orientation of neuronal fiber pathways and may facilitate surgical planning of patient-tailored approach (59). Many investigators have paid close attention to CST, also known as the pyramidal tract, which connects the sensorimotor cortex to the spinal cord to ensure extremities movement (4, 9, 11, 16, 82). Usually, BM with associated edema could cause a critical anterior displacement of the CST (9, 18), and dissection of the precentral, central, or postcentral sulcus is available and may therefore increase the safety of tumor resection.

**Figure 4:**
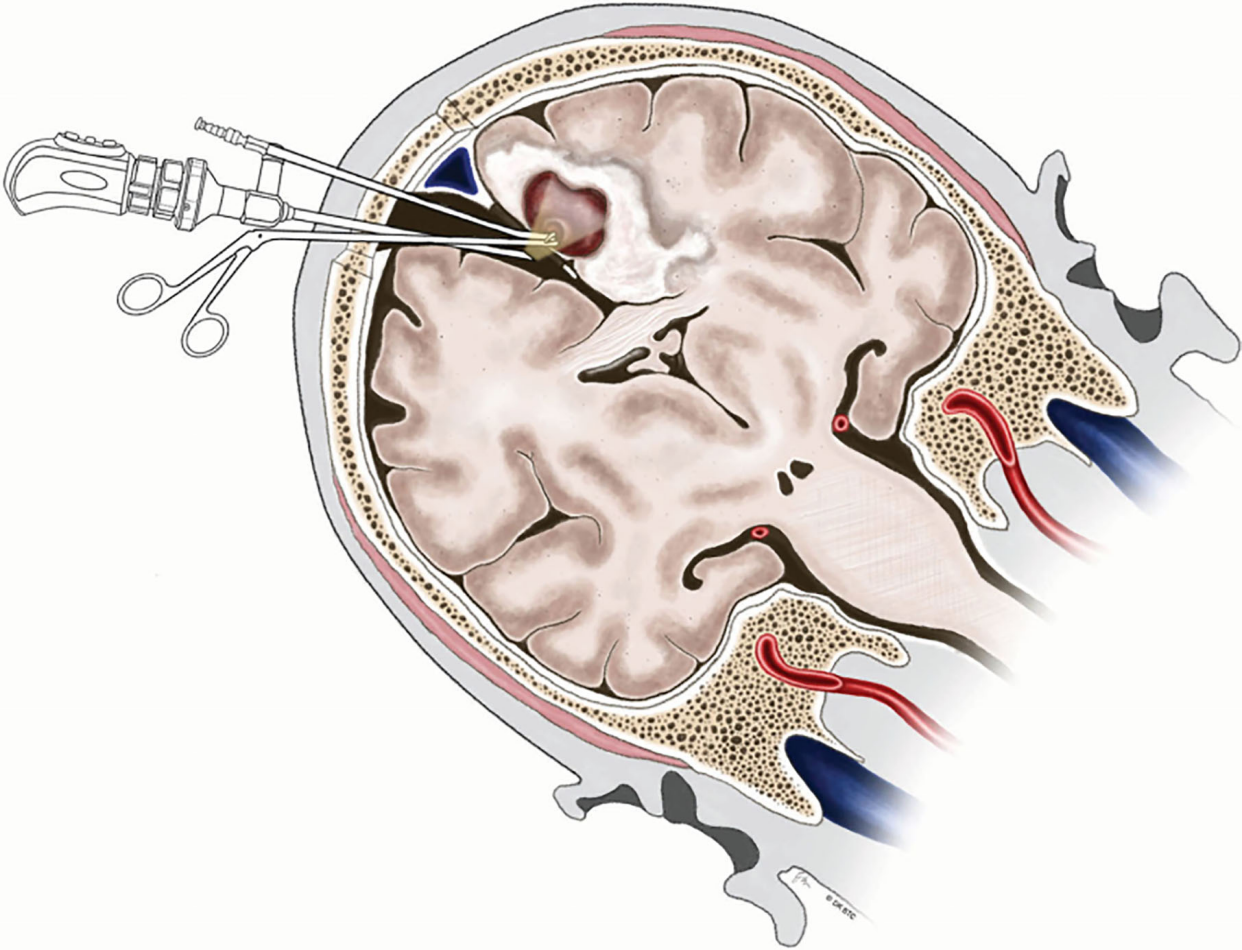
Illustration demonstrating endoscopy-assisted gravity-aided contralateral transfalcine approach for deep medial BM involving the paracentral lobule. BM, brain metastases. From Barkhoudarian et al. (76).

Deep medial BM involving the paracentral lobule could be reached by different approaches. Since Spetzler et al. (83) first described contralateral transcallosal approach in detail, contralateral interhemispheric transfalcine approach to parafalcine lesions has been widely used to provide better exposure avoiding invasion into the eloquent cortex and brain contusion (76, 84, 85). More inspiringly, the advent of endoscope has been proven to be useful in further enhancing the visualization of the deep-seated surgical cavity (76, 86). Barkhoudarian et al. (76) first took advantage of endoscopy-assisted gravity-aided contralateral transfalcine approach for deep para-midline BM within the paracentral lobule. After CSF release, the contralateral hemisphere falls away from midline to widen the interhemispheric space, which facilitates to open the falx and broadly expose the lesion without retraction of the vital cortex (**Figure 4**). Additionally, the endoscopic approach allows the surgeon to operate closer to the surgical area in more comfortable manner without fully extending the unsupported arms comparing with microscopic surgery (76).

## Future Perspectives

**Figure 5:**
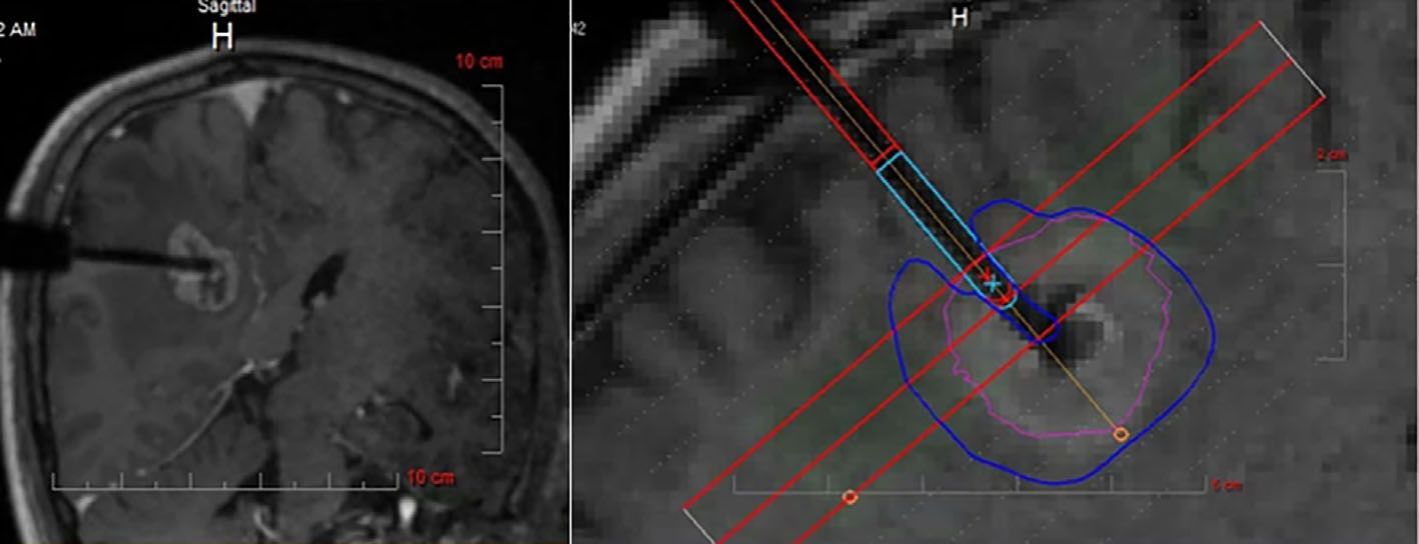
Intraoperative MRI monitoring of LITT after stereotactic placement of the laser electrode. The heat maps are presented showing temperature-dependent colors during treatment. MRI, magnetic resonance imaging; LITT, laser interstitial thermal therapy. Modified from Ferguson et al. (35).

Prevalence of BM involving the perirolandic region is increasing (9, 19). As previously reported, surgical removal can be used to promptly relieve the mass effect, improve clinical symptoms, obtain tumor tissue to identify novel makers for targeted or individualized therapies (35, 50, 51). Nowadays, patient-tailored treatment concept in BM has been widely accepted, including minimal invasiveness and multimodal therapy. Interstitial brachytherapy enables the accurate application of highly focused necrotizing tissue dose with a steep fall-off from the center to the periphery (9, 87), which can be indicated in eloquent BM that are not amenable to resection even after previous irradiation or radiosurgery (87, 88). Additionally, LITT can deliver enough thermal damage and induce coagulation to tumor while simultaneously avoiding damage to surrounding brain parenchyma (**Figure 5**) (35, 89, 90), shortening duration of both operation and hospitalization (91, 92). Lyer et al. first presented a patient who underwent MRI-guided LITT for BM in the motor strip and had an excellent outcome (66). After frozen biopsy, the laser fiber was placed down the planned track into the tumor bed which was then heated to 70 degrees for 3 min, resulting in the ablation length of 2.2 cm along the axis of the tumor. Although no other clinical data for LITT come from treating patients with BM affecting the perirolandic region, benefits and limitations of laser treatment compared with open surgery should be considered (**Table 3**), and ongoing formal investigations are continuing to determine the efficacy and indications for these therapies.

**Table 3:** Pros and cons of the LITT in comparison to open surgery in patients with BM.

Pros	Cons
Accurate real-time monitoring on navigation workstation Surgically inaccessible locations Tumors resistant to standard-of-care therapies such as surgical resection or SRT Minimally invasive procedure with less bleeding Reducing risk of infection due to thermal effect Lower incidence of complications Shorter operating times and length of stay, less medical expenses and time-consuming	Irregular lesions larger than 3 cm in diameter Refractory edema caused by tumors Seldom prompt symptom relief in patients with preoperative deficits Repeated process for multiple tumors

LITT, laser interstitial thermal therapy; BM, brain metastases; SRT, stereotactic radiotherapy.

BM in the perirolandic region may still carry a poor prognosis in spite of contemporary management and technical advances. Nevertheless, individualized and multimodal therapies have been identified as standard of care, and surgery still plays an important role as a cornerstone of therapy, which has been well accepted. More prospective randomized controlled studies are needed to determine if better local tumor control and improved overall survival could be achieved by more minimally invasive surgery.

## Author Contributions

FZ, JK, and YZ conceived and designed the study. FZ and KH collected the data from the previous literature. FZ analyzed the data and drafted the article. FZ and JW contributed to financial support. JW final approved the manuscript. All authors contributed to the article and approved the submitted version.

## Funding

This study was supported by the National Natural Science Foundation of China (No. 81701262) and the Beijing Hope Run Special Fund of Cancer Foundation of China (No. LC2017B13).

## Conflict of Interest

The authors declare that the research was conducted in the absence of any commercial or financial relationships that could be construed as a potential conflict of interest.

## Abbreviations

BM, brain metastases; WBRT, whole-brain radiation therapy; SRT, stereotactic radiotherapy; LITT, laser interstitial thermal therapy; MCA, middle cerebral artery; SSS, superior sagittal sinus; CST, corticospinal tract; fMRI, functional magnetic resonance imaging; 5-ALA, 5-aminolevulinic acid; FS, fluorescein sodium; GTR, gross-total resection; CT, computed tomography; DTI, diffusion tensor imaging; FA, fractional anisotropy; ADC, apparent diffusion coefficient; CSF, cerebrospinal fluid; SEPs, somatosensory evoked potentials; IONM, intraoperative neurophysiological monitoring; MEPs, motor evoked potentials; STR, subtotal resection; HF, high-frequency; LF, low-frequency; RPA, recursive partitioning analysis; RTOG, Radiation Therapy Oncology Group; KPS, Karnofsky Performance Score; MTR, microscopic total resection.

## References

[1] Frigeri T Paglioli E de Oliveira E Rhoton AL Jr. Microsurgical anatomy of the central lobe. J Neurosurg (2015) 122(3):483–98. 10.3171/2014.11.JNS14315 25555079

[2] Ribas GC The cerebral sulci and gyri. Neurosurg Focus (2010) 28(2):E2. 10.3171/2009.11.FOCUS09245 20121437

[3] Ostrom QT Wright CH Barnholtz-Sloan JS Brain metastases: epidemiology. Handb Clin Neurol (2018) 149:27–42. 10.1016/B978-0-12-811161-1.00002-5 29307358

[4] Sanmillan JL Fernandez-Coello A Fernandez-Conejero I Plans G Gabarros A Functional approach using intraoperative brain mapping and neurophysiological monitoring for the surgical treatment of brain metastases in the central region. J Neurosurg (2017) 126(3):698–707. 10.3171/2016.2.JNS152855 27128588

[5] Luther N Kondziolka D Kano H Mousavi SH Flickinger JC Lunsford LD Motor function after stereotactic radiosurgery for brain metastases in the region of the motor cortex. J Neurosurg (2013) 119(3):683–8. 10.3171/2013.6.JNS122081 23870018

[6] Walter J Kuhn SA Waschke A Kalff R Ewald C Operative treatment of subcortical metastatic tumours in the central region. J Neurooncol (2011) 103(3):567–73. 10.1007/s11060-010-0420-5 20878448

[7] Obermueller T Schaeffner M Gerhardt J Meyer B Ringel F Krieg SM Risks of postoperative paresis in motor eloquently and non-eloquently located brain metastases. BMC Cancer (2014) 14:21. 10.1186/1471-2407-14-21 24422871 PMC3899614

[8] Berghoff AS Rajky O Winkler F Bartsch R Furtner J Hainfellner JA Invasion patterns in brain metastases of solid cancers. Neuro Oncol (2013) 15(12):1664–72. 10.1093/neuonc/not112 PMC3829586 24084410

[9] Thon N Kreth FW Tonn JC The role of surgery for brain metastases from solid tumors. Handb Clin Neurol (2018) 149:113–21. 10.1016/B978-0-12-811161-1.00008-6 29307348

[10] Krieg SM Picht T Sollmann N Bahrend I Ringel F Nagarajan SS Resection of Motor Eloquent Metastases Aided by Preoperative nTMS-Based Motor Maps-Comparison of Two Observational Cohorts. Front Oncol (2016) 6:261. 10.3389/fonc.2016.00261 28066717 PMC5174728

[11] Lee SJ Hwang SC Im SB Kim BT Surgical Resection of Non-Glial Tumors in the Motor Cortex. Brain Tumor Res Treat (2016) 4(2):70–6. 10.14791/btrt.2016.4.2.70 PMC5114195 27867915

[12] Gaspar LE Mehta MP Patchell RA Burri SH Robinson PD Morris RE The role of whole brain radiation therapy in the management of newly diagnosed brain metastases: a systematic review and evidence-based clinical practice guideline. J Neurooncol (2010) 96(1):17–32. 10.1007/s11060-009-0060-9 19960231 PMC2808517

[13] Linskey ME Andrews DW Asher AL Burri SH Kondziolka D Robinson PD The role of stereotactic radiosurgery in the management of patients with newly diagnosed brain metastases: a systematic review and evidence-based clinical practice guideline. J Neurooncol (2010) 96(1):45–68. 10.1007/s11060-009-0073-4 19960227 PMC2808519

[14] Gaspar L Scott C Rotman M Asbell S Phillips T Wasserman T Recursive partitioning analysis (RPA) of prognostic factors in three Radiation Therapy Oncology Group (RTOG) brain metastases trials. Int J Radiat Oncol Biol Phys (1997) 37(4):745–51. 10.1016/s0360-3016(96)00619-0 9128946

[15] Mut M Surgical treatment of brain metastasis: a review. Clin Neurol Neurosurg (2012) 114(1):1–8. 10.1016/j.clineuro.2011.10.013 22047649

[16] Rossetto M Ciccarino P Lombardi G Rolma G Cecchin D Della Puppa A Surgery on motor area metastasis. Neurosurg Rev (2016) 39(1):71–7; discussion 7-8. 10.1007/s10143-015-0648-9 26178239

[17] Kalkanis SN Kondziolka D Gaspar LE Burri SH Asher AL Cobbs CS The role of surgical resection in the management of newly diagnosed brain metastases: a systematic review and evidence-based clinical practice guideline. J Neurooncol (2010) 96(1):33–43. 10.1007/s11060-009-0061-8 19960230 PMC2808516

[18] Krieg SM Schaffner M Shiban E Droese D Obermuller T Gempt J Reliability of intraoperative neurophysiological monitoring using motor evoked potentials during resection of metastases in motor-eloquent brain regions: clinical article. J Neurosurg (2013) 118(6):1269–78. 10.3171/2013.2.JNS121752 23521547

[19] Pintea B Baumert B Kinfe TM Gousias K Parpaley Y Bostrom JP Early motor function after local treatment of brain metastases in the motor cortex region with stereotactic radiotherapy/radiosurgery or microsurgical resection: a retrospective study of two consecutive cohorts. Radiat Oncol (2017) 12(1):177. 10.1186/s13014-017-0917-6 29132382 PMC5683312

[20] Lischalk JW Oermann E Collins SP Nair MN Nayar VV Bhasin R Five-fraction stereotactic radiosurgery (SRS) for single inoperable high-risk non-small cell lung cancer (NSCLC) brain metastases. Radiat Oncol (2015) 10:216. 10.1186/s13014-015-0525-2 26503609 PMC4624578

[21] Metellus P Bialecki E Le Rhun E Dhermain F Neurosurgical and radiosurgical decision making in brain metastasis patients in the area of targeted therapies? Chin Clin Oncol (2015) 4(2):19. 10.3978/j.issn.2304-3865.2015.06.02 26112805

[22] Kocher M Soffietti R Abacioglu U Villa S Fauchon F Baumert BG Adjuvant whole-brain radiotherapy versus observation after radiosurgery or surgical resection of one to three cerebral metastases: results of the EORTC 22952-26001 study. J Clin Oncol (2011) 29(2):134–41. 10.1200/JCO.2010.30.1655 PMC3058272 21041710

[23] Williams BJ Suki D Fox BD Pelloski CE Maldaun MV Sawaya RE Stereotactic radiosurgery for metastatic brain tumors: a comprehensive review of complications. J Neurosurg (2009) 111(3):439–48. 10.3171/2008.11.JNS08984 19301968

[24] Shinoura N Yoshida M Yamada R Tabei Y Saito K Suzuki Y Awake surgery with continuous motor testing for resection of brain tumors in the primary motor area. J Clin Neurosci (2009) 16(2):188–94. 10.1016/j.jocn.2008.02.013 19071024

[25] Romstock J Fahlbusch R Ganslandt O Nimsky C Strauss C Localisation of the sensorimotor cortex during surgery for brain tumours: feasibility and waveform patterns of somatosensory evoked potentials. J Neurol Neurosurg Psychiatry (2002) 72(2):221–9. 10.1136/jnnp.72.2.221 PMC1737735 11796773

[26] Kellogg RG Munoz LF Selective excision of cerebral metastases from the precentral gyrus. Surg Neurol Int (2013) 4:66. 10.4103/2152-7806.112189 23776752 PMC3683173

[27] Weil RJ Lonser RR Selective excision of metastatic brain tumors originating in the motor cortex with preservation of function. J Clin Oncol (2005) 23(6):1209–17. 10.1200/JCO.2005.04.124 15718318

[28] Gempt J Krieg SM Huttinger S Buchmann N Ryang YM Shiban E Postoperative ischemic changes after glioma resection identified by diffusion-weighted magnetic resonance imaging and their association with intraoperative motor evoked potentials. J Neurosurg (2013) 119(4):829–36. 10.3171/2013.5.JNS121981 23829818

[29] Rhoton AL Jr. The cerebral veins. Neurosurgery (2002) 51(4 Suppl):S159–205. 10.1097/00006123-200210001-00005 12234449

[30] Farrell DF Burbank N Lettich E Ojemann GA Individual variation in human motor-sensory (rolandic) cortex. J Clin Neurophysiol (2007) 24(3):286–93. 10.1097/WNP.0b013e31803bb59a 17545834

[31] Patel AJ Suki D Hatiboglu MA Rao VY Fox BD Sawaya R Impact of surgical methodology on the complication rate and functional outcome of patients with a single brain metastasis. J Neurosurg (2015) 122(5):1132–43. 10.3171/2014.9.JNS13939 25794344

[32] Yoo H Kim YZ Nam BH Shin SH Yang HS Lee JS Reduced local recurrence of a single brain metastasis through microscopic total resection. J Neurosurg (2009) 110(4):730–6. 10.3171/2008.8.JNS08448 19072310

[33] Ghosh M MK Trivedi V Chauhan R Shubham S Muneer A Clinical profle of patients with brain metastasis - a single institutional retrospective study. Int J Contemp Med Res (2017) 4(2):372–6.

[34] Chukwueke UN Brastianos PK Precision Medical Approaches to the Diagnoses and Management of Brain Metastases. Curr Treat Options Oncol (2019) 20(6):49. 10.1007/s11864-019-0649-y 31062107

[35] Ferguson SD Wagner KM Prabhu SS McAleer MF McCutcheon IE Sawaya R Neurosurgical management of brain metastases. Clin Exp Metastasis (2017) 34(6-7):377–89. 10.1007/s10585-017-9860-z 28965270

[36] Lin NU Lee EQ Aoyama H Barani IJ Barboriak DP Baumert BG Response assessment criteria for brain metastases: proposal from the RANO group. Lancet Oncol (2015) 16(6):e270–8. 10.1016/S1470-2045(15)70057-4 26065612

[37] Black PM Johnson MD Surgical resection for patients with solid brain metastases: current status. J Neurooncol (2004) 69(1-3):119–24. 10.1023/b:neon.0000041875.09048.e7 15527084

[38] Li J Bentzen SM Renschler M Mehta MP Regression after whole-brain radiation therapy for brain metastases correlates with survival and improved neurocognitive function. J Clin Oncol (2007) 25(10):1260–6. 10.1200/JCO.2006.09.2536 17401015

[39] Kamp MA Dibue M Niemann L Reichelt DC Felsberg J Steiger HJ Proof of principle: supramarginal resection of cerebral metastases in eloquent brain areas. Acta Neurochir (Wien) (2012) 154(11):1981–6. 10.1007/s00701-012-1463-5 22875595

[40] Kamp MA Rapp M Slotty PJ Turowski B Sadat H Smuga M Incidence of local in-brain progression after supramarginal resection of cerebral metastases. Acta Neurochir (Wien) (2015) 157(6):905–10; discussion 10-1. 10.1007/s00701-015-2405-9 25845550

[41] Kamp MA Grosser P Felsberg J Slotty PJ Steiger HJ Reifenberger G 5-aminolevulinic acid (5-ALA)-induced fluorescence in intracerebral metastases: a retrospective study. Acta Neurochir (Wien) (2012) 154(2):223–8; discussion 8. 10.1007/s00701-011-1200-5 22080159

[42] Baumert BG Rutten I Dehing-Oberije C Twijnstra A Dirx MJ Debougnoux-Huppertz RM A pathology-based substrate for target definition in radiosurgery of brain metastases. Int J Radiat Oncol Biol Phys (2006) 66(1):187–94. 10.1016/j.ijrobp.2006.03.050 16814946

[43] Siam L Bleckmann A Chaung HN Mohr A Klemm F Barrantes-Freer A The metastatic infiltration at the metastasis/brain parenchyma-interface is very heterogeneous and has a significant impact on survival in a prospective study. Oncotarget (2015) 6(30):29254–67. 10.18632/oncotarget.4201 PMC4745724 26299612

[44] Sundaresan N Galicich JH Surgical treatment of brain metastases. Clinical and computerized tomography evaluation of the results of treatment. Cancer (1985) 55(6):1382–8. 10.1002/1097-0142(19850315)55:6<1382::aid-cncr2820550637>3.0.co;2-z 3971308

[45] Plataniotis GA Theofanopoulou M Sotiriadou K Vlychou M Fountoulis G Fezoulidis J The volume of brain metastases may be of prognostic significance in patients with non-small-cell lung cancer classified as RTOG-RPA classes 2 and 3. Clin Oncol (R Coll Radiol) (2006) 18(1):85–6. 10.1016/j.clon.2005.07.013 16477927

[46] Andrews RJ Gluck DS Konchingeri RH Surgical resection of brain metastases from lung cancer. Acta Neurochir (Wien) (1996) 138(4):382–9. 10.1007/BF01420299 8738387

[47] Mintz AP Cairncross JG Treatment of a single brain metastasis: the role of radiation following surgical resection. JAMA (1998) 280(17):1527–9. 10.1001/jama.280.17.1527 9809735

[48] Akanda ZZ Hong W Nahavandi S Haghighi N Phillips C Kok DL Post-operative stereotactic radiosurgery following excision of brain metastases: A systematic review and meta-analysis. Radiother Oncol (2020) 142:27–35. 10.1016/j.radonc.2019.08.024 31563407

[49] Arvold ND Lee EQ Mehta MP Margolin K Alexander BM Lin NU Updates in the management of brain metastases. Neuro Oncol (2016) 18(8):1043–65. 10.1093/neuonc/now127 PMC4933491 27382120

[50] Morikawa A Peereboom DM Thorsheim HR Samala R Balyan R Murphy CG Capecitabine and lapatinib uptake in surgically resected brain metastases from metastatic breast cancer patients: a prospective study. Neuro Oncol (2015) 17(2):289–95. 10.1093/neuonc/nou141 PMC4288517 25015089

[51] Bartsch R Berghoff AS Preusser M Breast cancer brain metastases responding to primary systemic therapy with T-DM1. J Neurooncol (2014) 116(1):205–6. 10.1007/s11060-013-1257-5 24065570

[52] Della Puppa A De Pellegrin S d’Avella E Gioffre G Rossetto M Gerardi A 5-aminolevulinic acid (5-ALA) fluorescence guided surgery of high-grade gliomas in eloquent areas assisted by functional mapping. Our experience and review of the literature. Acta Neurochir (Wien) (2013) 155(6):965–72; discussion 72. 10.1007/s00701-013-1660-x 23468036

[53] Miner RC Image-Guided Neurosurgery. J Med Imaging Radiat Sci (2017) 48(4):328–35. 10.1016/j.jmir.2017.06.005 31047466

[54] Schebesch KM Hoehne J Hohenberger C Proescholdt M Riemenschneider MJ Wendl C Fluorescein sodium-guided resection of cerebral metastases-experience with the first 30 patients. Acta Neurochir (Wien) (2015) 157(6):899–904. 10.1007/s00701-015-2395-7 25824557

[55] Hohne J Hohenberger C Proescholdt M Riemenschneider MJ Wendl C Brawanski A Fluorescein sodium-guided resection of cerebral metastases-an update. Acta Neurochir (Wien) (2017) 159(2):363–7. 10.1007/s00701-016-3054-3 28012127

[56] Policicchio D Doda A Sgaramella E Ticca S Veneziani Santonio F Boccaletti R Ultrasound-guided brain surgery: echographic visibility of different pathologies and surgical applications in neurosurgical routine. Acta Neurochir (Wien) (2018) 160(6):1175–85. 10.1007/s00701-018-3532-x 29675718

[57] Barbagallo G Maione M Peschillo S Signorelli F Visocchi M Sortino G Intraoperative Computed Tomography, navigated ultrasound, 5-Amino-Levulinic Acid fluorescence and neuromonitoring in brain tumor surgery: overtreatment or useful tool combination? J Neurosurg Sci. 10.23736/S0390-5616.19.04735-0 31298506

[58] Rutten GJ Ramsey NF The role of functional magnetic resonance imaging in brain surgery. Neurosurg Focus (2010) 28(2):E4. 10.3171/2009.12.FOCUS09251 20121439

[59] Krieg SM Shiban E Buchmann N Gempt J Foerschler A Meyer B Utility of presurgical navigated transcranial magnetic brain stimulation for the resection of tumors in eloquent motor areas. J Neurosurg (2012) 116(5):994–1001. 10.3171/2011.12.JNS111524 22304452

[60] Krivosheya D Rao G Tummala S Kumar V Suki D Bastos DCA Impact of Multi-modality Monitoring Using Direct Electrical Stimulation to Determine Corticospinal Tract Shift and Integrity in Tumors using the Intraoperative MRI. J Neurol Surg A Cent Eur Neurosurg (2019). 10.1055/s-0039-1698383 31659724

[61] D’Andrea G Angelini A Romano A Di Lauro A Sessa G Bozzao A Intraoperative DTI and brain mapping for surgery of neoplasm of the motor cortex and the corticospinal tract: our protocol and series in BrainSUITE. Neurosurg Rev (2012) 35(3):401–12; discussion 12. 10.1007/s10143-012-0373-6 22370809

[62] Bobek-Billewicz B Stasik-Pres G Majchrzak K Senczenko W Majchrzak H Jurkowski M Fibre integrity and diffusivity of the pyramidal tract and motor cortex within and adjacent to brain tumour in patients with or without neurological deficits. Folia Neuropathol (2011) 49(4):262–70. 22212916

[63] Martino J Gabarros A Deus J Juncadella M Acebes JJ Torres A Intrasurgical mapping of complex motor function in the superior frontal gyrus. Neuroscience (2011) 179:131–42. 10.1016/j.neuroscience.2011.01.047 21277357

[64] Garber ST Jensen RL Image guidance for brain metastases resection. Surg Neurol Int (2012) 3(Suppl 2):S111–7. 10.4103/2152-7806.95422 PMC3400496 22826814

[65] Nabavi A Black PM Gering DT Westin CF Mehta V Pergolizzi RS Jr. Serial intraoperative magnetic resonance imaging of brain shift. Neurosurgery (2001) 48(4):787–97. 10.1097/00006123-200104000-00019 11322439

[66] Iyer A Halpern CH Grant GA Deb S Li GH Magnetic Resonance-Guided Laser-Induced Thermal Therapy for Recurrent Brain Metastases in the Motor Strip After Stereotactic Radiosurgery. Cureus (2016) 8(12):e919. 10.7759/cureus.919 28083463 PMC5218883

[67] Penfeld W BE Somatic motor and sensory representation in the cerebral cortex of man as studied by electrical stimulation. Brain (1937) 60:389–443. 10.1093/brain/60.4.389

[68] Macdonald DB Skinner S Shils J Yingling C American Society of Neurophysiological M. Intraoperative motor evoked potential monitoring - a position statement by the American Society of Neurophysiological Monitoring. Clin Neurophysiol (2013) 124(12):2291–316. 10.1016/j.clinph.2013.07.025 24055297

[69] Duffau H Capelle L Sichez J Faillot T Abdennour L Law Koune JD Intra-operative direct electrical stimulations of the central nervous system: the Salpetriere experience with 60 patients. Acta Neurochir (Wien) (1999) 141(11):1157–67. 10.1007/s007010050413 10592115

[70] Obermueller T Schaeffner M Shiban E Droese D Negwer C Meyer B Intraoperative neuromonitoring for function-guided resection differs for supratentorial motor eloquent gliomas and metastases. BMC Neurol (2015) 15:211–21. 10.1186/s12883-015-0476-0 PMC4618356 26487091

[71] Rossi M Conti Nibali M Viganò L Puglisi G Howells H Gay L Resection of tumors within the primary motor cortex using high-frequency stimulation: oncological and functional efficiency of this versatile approach based on clinical conditions. J Neurosurg (2020) 133:642–54. 10.3171/2019.5.jns19453 31398706

[72] Bander ED Shelkov E Modik O Kandula P Karceski SC Ramakrishna R Use of the train-of-five bipolar technique to provide reliable, spatially accurate motor cortex identification in asleep patients. Neurosurg Focus (2020) 48(2):E4. 10.3171/2019.11.focus19776 32006941

[73] Schackert G Steinmetz A Meier U Sobottka SB Surgical management of single and multiple brain metastases: results of a retrospective study. Onkologie (2001) 24(3):246–55. 10.1159/000055087 11455217

[74] Tan TC Mc LBP Image-guided craniotomy for cerebral metastases: techniques and outcomes. Neurosurgery (2003) 53(1):82–9; discussion 9-90. 10.1227/01.neu.0000068729.37362.f9 12823876

[75] Tobler WD Stanley M Stereotactic resection of brain metastases in eloquent brain. Stereotact Funct Neurosurg (1994) 63(1-4):38–44. 10.1159/000100289 7624649

[76] Barkhoudarian G Farahmand D Louis RG Oksuz E Sale D Villanueva P Microsurgical Endoscope-Assisted Gravity-Aided Transfalcine Approach for Contralateral Metastatic Deep Medial Cortical Tumors. Oper Neurosurg (Hagerstown) (2017) 13(6):724–31. 10.1093/ons/opx067 29186601

[77] Suki D Hatiboglu MA Patel AJ Weinberg JS Groves MD Mahajan A Comparative risk of leptomeningeal dissemination of cancer after surgery or stereotactic radiosurgery for a single supratentorial solid tumor metastasis. Neurosurgery (2009) 64(4):664–74; discussion 74-6. 10.1227/01.NEU.0000341535.53720.3E 19197219

[78] Suki D Abouassi H Patel AJ Sawaya R Weinberg JS Groves MD Comparative risk of leptomeningeal disease after resection or stereotactic radiosurgery for solid tumor metastasis to the posterior fossa. J Neurosurg (2008) 108(2):248–57. 10.3171/JNS/2008/108/2/0248 18240919

[79] Patel AJ Suki D Hatiboglu MA Abouassi H Shi W Wildrick DM Factors influencing the risk of local recurrence after resection of a single brain metastasis. J Neurosurg (2010) 113(2):181–9. 10.3171/2009.11.JNS09659 20035574

[80] Kronfeld-Duenias V Amir O Ezrati-Vinacour R Civier O Ben-Shachar M The frontal aslant tract underlies speech fluency in persistent developmental stuttering. Brain Struct Funct (2016) 221(1):365–81. 10.1007/s00429-014-0912-8 25344925

[81] Chang EF Raygor KP Berger MS Contemporary model of language organization: an overview for neurosurgeons. J Neurosurg (2015) 122(2):250–61. 10.3171/2014.10.JNS132647 25423277

[82] Natali AL Reddy V Bordoni B Neuroanatomy, Corticospinal Cord Tract. (Treasure Island, FL: StatPearls) (2020). 30571044

[83] Lawton MT Golfinos JG Spetzler RF The contralateral transcallosal approach: experience with 32 patients. Neurosurgery (1996) 39(4):729–34; discussion 34-5. 10.1097/00006123-199610000-00016 8880765

[84] Malekpour M Cohen-Gadol AA Interhemispheric transfalcine approach and awake cortical mapping for resection of peri-atrial gliomas associated with the central lobule. J Clin Neurosci (2015) 22(2):383–6. 10.1016/j.jocn.2014.07.017 25304435

[85] Kim YB Young WL Lawton MT Project UBS Parafalcine and midline arteriovenous malformations: surgical strategy, techniques, and outcomes. J Neurosurg (2011) 114(4):984–93. 10.3171/2010.12.JNS101297 21250805

[86] Plaha P Livermore LJ Voets N Pereira E Cudlip S Minimally invasive endoscopic resection of intraparenchymal brain tumors. World Neurosurg (2014) 82(6):1198–208. 10.1016/j.wneu.2014.07.034 25084167

[87] Schwarz SB Thon N Nikolajek K Niyazi M Tonn JC Belka C Iodine-125 brachytherapy for brain tumours–a review. Radiat Oncol (2012) 7:30. 10.1186/1748-717X-7-30 22394548 PMC3354996

[88] Romagna A Schwartz C Egensperger R Watson J Tonn JC Belka C Iodine-125 brachytherapy as upfront and salvage treatment for brain metastases : A comparative analysis. Strahlenther Onkol (2016) 192(11):780–8. 10.1007/s00066-016-1009-5 27349709

[89] Lagman C Chung LK Pelargos PE Ung N Bui TT Lee SJ Laser neurosurgery: A systematic analysis of magnetic resonance-guided laser interstitial thermal therapies. J Clin Neurosci (2017) 36:20–6. 10.1016/j.jocn.2016.10.019 27838155

[90] Medvid R Ruiz A Komotar RJ Jagid JR Ivan ME Quencer RM Current Applications of MRI-Guided Laser Interstitial Thermal Therapy in the Treatment of Brain Neoplasms and Epilepsy: A Radiologic and Neurosurgical Overview. AJNR Am J Neuroradiol (2015) 36(11):1998–2006. 10.3174/ajnr.A4362 26113069 PMC7964876

[91] Carpentier A McNichols RJ Stafford RJ Itzcovitz J Guichard JP Reizine D Real-time magnetic resonance-guided laser thermal therapy for focal metastatic brain tumors. Neurosurgery (2008) 63(1 Suppl 1):ONS21–8; discussion ONS8-9. 10.1227/01.neu.0000335007.07381.df 18728600

[92] Salem U Kumar VA Madewell JE Schomer DF De Almeida Bastos DC Zinn PO Neurosurgical applications of MRI guided laser interstitial thermal therapy (LITT). Cancer Imaging (2019) 19:65–77. 10.1186/s40644-019-0250-4 31615562 PMC6792239

